# Some Notes on Quantum Information in Spacetime

**DOI:** 10.3390/e22080864

**Published:** 2020-08-06

**Authors:** Ignazio Licata

**Affiliations:** 1 ISEM, Institute for Scientific Methodology, 90121 Palermo, Italy; ignazio.licata3@gmail.com; 2 School of Advanced International Studies on Applied Theoretical and Non-LinearMethodologies in Physics, 70121 Bari, Italy

The results obtained since the 70s with the study of Hawking radiation and the Unruh effect have highlighted a new domain of authority of relativistic principles. Entanglement, the quantum phenomenon par excellence, is in fact observer dependent [1], and the very concept of “particle” does not have the same information content for different observers [2,3]. All this proposes the centrality of the notion of “event” in physics and the meaning of its informational value. It is in this direction that Quantum Relativistic Information (QRI) is defined, which can therefore be defined as the study of quantum states in a relational context.

It must be said that, despite being a prelude to a future quantum gravity, QRI is a largely autonomous field—because it does not imply any specific hypothesis on the Planck scale—and is characterized by some principles that guard an assumption of great epistemological strength. As A. Zeilinger [4] says, it is impossible to distinguish between “reality” and “description of reality”, i.e., information in the study of physics; doing so means jeopardizing the universal value and beauty of physical laws. Both relativity and quantum physics are aspects of a broader information theory that we have been discovering in recent years and within which the foundational debate is renewed with new experimental possibilities. The first principle we need is therefore:

The principle of contextuality [5]: Each description of a class of events must contain, implicitly or explicitly, the reference structure of the observer. In other words, it must be possible for each observer to define assign values for each observable.

A very strong request comes from the principle of equivalence, which, after showing unsuspected resistance to any attempt of de-construction, is now extended to the quantum domain as a request to describe gravitational phenomena in terms of causal networks [6,7,8,9,10,11]. L. Susskind and G. ’t Hooft proposal for the information paradox adds a new element to the picture: the complementarity invoked is in fact a principle of equivalence [12,13]. Although the Black Holes question are still far from being resolved (with particular regard to the core of the BH, with interesting inter-connections between strings, non-commutativity and euclidicity, see for example: [14,15,16,17,18,19,20]), the synthesis of equivalence and complementarity leads to a powerful holographic principle that introduces, according to Bekenstein’s limit [21], a new way of looking at the locality and a different approach to cosmology. The holographic principle feeds on conjectures and is still looking for theories (duality between gravity and quantum field theory: [22,23,24,25,26]), but it is a catalyst for new conceptual suggestions regarding the physical meaning of the cosmological horizon. In particular, considering the four-dimensional dynamics as the explication (in a Bohmian sense) of a De Sitter non-perturbative vacuum offers an improvement of Hartle–Hawking proposal in quantum cosmology and a solution to the informational paradox in the BH [27,28,29]. This line of reasoning is also promising for an event-based reading of Quantum Mechanics [30].

For a long time, holography and emergentism appeared as two styles of explanation irreconcilable with respect to the locality, but an emergency of time could offer new perspectives with a duality between imaginary time and real time, in a diachronic/synchronic complementarity [31,32,33].

It is known that there are well-defined whormhole solutions in General Relativity and Yang Mills Theory, and the recent ER = EPR conjecture proposes the question of the emergence of metric space-time from a non-local background [34,35,36,37,38]. A suggestion in the direction of the laboratory comes from the Bose–Marletto–Vedral conjecture on the possible coalescence of two quantum systems in a non-local phase, which would reveal the limits of the local metric description and the non-classical aspects of space-time [39,40]. A covariant analysis of this situation shows that discrete effects could prove to be an overlap of geometries measurable through entanglement entropy [41,42].

Furthermore, localization appears as the production of a new degree of freedom. We assume, in accordance with a recent proposal [30,43], that the localization R of a process is associated with the genesis of a micro-horizon of de Sitter of center O and radius cθ0 ≈ 10–13 cm (chronon, corresponding to the classical radius of the electron), with O generally delocalized according to the wave function entering/leaving the process. The constant θ0 is independent of cosmic time, so the ratio t0/θ0 ≈ 10^41^ is also independent of cosmic time, with ct0 ≈ 1028 cm. This ratio expresses the number of totally distinct temporal locations accessible by the R process within the horizon of cosmological de Sitter. In practice, the time line segment on which an observer at the center of the horizon places the process R has length t0, while the duration of the process R is in the order of θ0; the segment is therefore divided into separate t0/θ0 ≈ 10^41^ “cells”. Each cell can be in two states: “on” or “off”. The temporal localization of a single process R corresponds to the situation in which all the cells are switched off minus one. Configurations with multiple cells on will correspond to the location of multiple distinct R processes on the same time line. If you accept the idea that each cell is independent, you have $2^{10^{41}}$ distinct configurations in all. The positional information associated with the location of 0, 1, 2, …, 10^41^ R processes then amounts to 1041 bits, the binary logarithm of the number of configurations. This is a kind of coded information on the time axis contained within the observer’s de Sitter horizon.

The R processes are in fact real interactions between real particles, during which an amount of action is exchanged in the order of the Planck quantum h. Therefore, in terms of phase space, the manifestation of one of these processes is equivalent to the ignition of an elementary cell of volume h3. The number of “switched on” cells in the phase space of a given macroscopic physical system is an estimator of the volume it occupies in this space, and therefore of its entropy. It is therefore conceivable that the location information of the R processes is connected to entropy through the uncertainty principle. This possibility presupposes the “objective” nature of the R processes.

It is therefore natural to ask whether some form of Bekenstein’s limit on entropy applies in some way to the two horizons mentioned. If we assume that the information on the temporal location of the processes R, I = 10^41^ bits, is connected to the area of the micro-horizon, A = (cθ0) 2 ≈ 10^−26^ cm^2^ from the holographic relationship:

(1) $$\frac{A}{4l^{2}}=I$$

Then, the spatial extension *l* of the “cells” associated with an information bit is ≈10^−33^ cm, the Planck scale! It is necessary to underline that the Planck scale presents itself in this way as a consequence of the holographic conjecture (1), combined with the “two horizons” hypothesis, and therefore of the finiteness of the information I. It in no way represents a limit to the continuity of spacetime, nor to the spatial or temporal distance between two events (which remains a continuous variable). Furthermore, since I = t0/θ0 and t0 is related to the cosmological constant λ by the relation λ = 4/3t02, the (1) is essentially a definition of the Planck scale as a function of the cosmological constant. A global-local relationship is exactly what we expect from a holographic vacuum theory.

## References

[1] Alsing P.M., Fuentes I. (2012). Observer dependent entanglement, Class. Quantum Grav..

[2] Davies P.C.W., Christensen S.M. (1984). Particles do not exist. Quantum Theory of Gravity.

[3] Colosi D., Rovelli C. (2009). What is a particle?. Class. Quant. Grav..

[4] Zeilinger A. (2010). Dance of the Photons: From Einstein to Quantum Teleportation.

[5] Jaroszkiewicz G., Licata I. (2016). Observers and Reality, in Beyond Peaceful Coexistence. The Emergence of Space, Time and Quantum.

[6] Licata I., Corda C., Benedetto E. (2016). A machian request for the equivalence principle in extended gravity and non-geodesic motion. Grav. Cosmol..

[7] Licata I., Benedetto E. (2018). The Charge in a Lift. A Covariance Problem. Gravit. Cosmol..

[8] Candelas P., Sciama D.W. (1983). Is there a quantum equivalence principle?. Phys. Rev. D.

[9] Tamburini F., de Laurentis M.F., Licata I. (2018). Radiation from charged particles due to explicit symmetry breaking in a gravitational field. Int. J. Geom. Methods Mod. Phys..

[10] Zych M., Brukner C. (2018). Quantum formulation of the Einstein equivalence principle. Nat. Phys..

[11] Hardy L. (2019). Implementation of the Quantum Equivalence Principle. arXiv.

[12] Hooft G. (1993). Dimensional Reduction in Quantum Gravity. arXiv.

[13] Susskind L. (2006). The paradox of quantum black holes. Nat. Phys..

[14] Susskind L. (1995). String Physics and Black Holes. Nucl. Phys. Proc. Suppl..

[15] Strominger A., Vafa C. (1996). Microscopic Origin of the Bekenstein-Hawking Entropy. Phys. Lett..

[16] Yogendran K.P. (2015). Horizon strings and interior states of a black hole. Phys. Lett..

[17] Nicolini P. (2009). Noncommutative Black Holes. The Final Appeal to Quantum Gravity: A Review. Int. J. Mod. Phys..

[18] Dowker F., Gregory R., Traschen J. (1992). Euclidean Black Hole Vortices. Phys. Rev..

[19] Hirayama T., Holdom B. (2003). Can black holes have Euclidean cores?. Phys. Rev..

[20] Corda C. (2013). Black hole quantum spectrum. Eur. Phys. J..

[21] Bekenstein J., Schiffer M. (1990). Quantum Limitations on the Storage and Transmission of Information. Int. J. Mod. Phys..

[22] Bousso R. (2002). The Holographic principle. Rev. Mod. Phys..

[23] Susskind L. (1995). The world as an hologram. J. Math. Phys..

[24] Maldacena J.M. (1998). The Large N Limit of Superconformal Field Theories and Supergravity. Adv. Theor. Math. Phys..

[25] Witten E. (1998). Anti De Sitter Space and Holography. Adv. Theor. Math. Phys..

[26] Dong X., Silverstein E., Torroba G. (2018). De Sitter holography and entanglement entropy. J. High Energy Phys..

[27] Nikolić H. (2009). Resolving the black-hole information paradox by treating time on an equal footing with space. Phys. Lett. B.

[28] Feleppa F., Licata I., Corda C. (2019). Hartle-Hawking boundary conditions as Nucleation by de Sitter Vacuum. Phys. Dark Universe.

[29] Licata I., Fiscaletti D., Chiatti L., Tamburini F., Davide F. (2020). CPT symmetry in cosmology and the Archaic Universe. Phys. Scr..

[30] Licata I., Chiatti L. (2019). Event-Based Quantum Mechanics: A Context for the Emergence of Classical Information. Symmetry.

[31] Vistarini T. (2017). Holographic space and time: Emergent in what sense?. Stud. Hist. Philos. Sci. Part B: Stud. Hist. Philos. Mod. Phys..

[32] Crowther K. (2020). As below, so before: ‘synchronic’ and ‘diachronic’ conceptions of spacetime emergence. Synthese.

[33] Licata I. (2016). In and Out of the Screen. On some new considerations about localization and delocalization in Archaic Theory, in Beyond Peaceful Coexistence. The Emergence of Space, Time and Quantum.

[34] Kim H. (1998). Classical and quantum wormholes in Einstein-Yang-Mills theory. Nucl. Phys. B.

[35] Maldacena J., Susskind L. (2013). Cool horizons for entangled black holes. Fortsch. Phys..

[36] Susskind L. (2016). Copenhagen vs Everett, Teleportation, and ER = EPR. Fortschr. Phys..

[37] Cao C., Carroll S., Michalakis S. (2017). Space from Hilbert Space: Recovering Geometry from Bulk Entanglement. Phys. Rev..

[38] Tamburini F., Licata I. (2019). General Relativistic Wormhole Connections from Planck-Scales and the ER = EPR Conjecture. Entropy.

[39] Bose S., Mazumdar A., Morley G.W., Ulbricht H., Toroš M., Paternostro M., Geraci A.A., Barker P.F., Kim M.S., Milburn G. (2017). Spin Entanglement Witness for Quantum Gravity. Phys. Rev. Lett..

[40] Marletto C., Vedral V. (2017). Gravitationally Induced Entanglement between Two Massive Particles is Sufficient Evidence of Quantum Effects in Gravity. Phys. Rev. Lett..

[41] Christodoulou M., Rovelli C. (2019). On the possibility of laboratory evidence for quantum superposition of geometries. Phys. Lett. B.

[42] Giacomini F., Castro-Ruiz E., Brukner C. (2019). Quantum mechanics and the covariance of physical laws in quantum reference frames. Nat. Commun..

[43] Chiatti L., Licata I. (2016). Particle model from quantum foundations. Quantum Stud. Math. Found..

